# Systematisches Risikomanagement für eine geplante Gasabschaltung im High-Care-Bereich eines Universitätsklinikums

**DOI:** 10.1007/s00101-023-01254-8

**Published:** 2023-02-08

**Authors:** Axel R. Heller, Maria Eberlein-Gonska, Hanns C. Held, Thea Koch

**Affiliations:** 1 Klinik für Anästhesiologie und Operative Intensivmedizin, Universitätsklinikum Augsburg, Stenglinstraße 2, 86156 Augsburg, Deutschland; 2 Klinik und Poliklinik für Anästhesiologie und Intensivmedizin, Universitätsklinikum Dresden, Dresden, Deutschland; 3 medizinischer Katastrophenschutzbeauftragter, Universitätsklinikum Dresden, Dresden, Deutschland; 4 Qualitäts- und Medizinisches Risikomanagement, Universitätsklinikum Dresden, Dresden, Deutschland; 5 Klinik und Poliklinik für Viszeral‑, Thorax- und Gefäßchirurgie, Universitätsklinikum Dresden, Dresden, Deutschland

**Keywords:** Krankenhausalarm- und Krankenhauseinsatzplanung, Beatmung, Intensivmedizin, Sauerstoff, Druckluft, Hospital emergency plan, Mechanical ventilation, Intensive care, Oxygen, Compressed air

## Abstract

**Hintergrund:**

Im Rahmen von Erweiterungsbaumaßnahmen am Uniklinikum Dresden war die Abschaltung der zentralen medizinischen Gasversorgung in einem Gebäude mit 3 intensivmedizinischen Teilstationen mit 22 Betten, einem OP-Trakt mit 5 OP und 6 Normalstationen mit je 28 Betten im laufenden Betrieb erforderlich. Damit bestand der Bedarf für die betroffenen Funktionseinheiten, für die Baumaßnahme eine interimistische dezentrale Gasversorgung mit Nullfehlertoleranz zu schaffen.

**Methodik:**

Nach etablierten Verfahren der Risiko- und Fehlermöglichkeitsanalyse wurde durch den Notfall- und Katastrophenschutzbeauftragten des Klinikums eine Projektgruppe ins Leben gerufen, die einen Projektplan, eine Bedarfsabschätzung und einen Kommunikationsplan erarbeitete.

**Ergebnisse:**

Eine Vielzahl von Risikofaktoren, für die geeignete Gegenmaßnahmen zu konzipieren waren, wurde systematisch ermittelt. Die Bedarfsabschätzung auf Basis physiologischer Parameter für die maximal 22 zu belegenden Beatmungsplätze über 4 h ergab je 26.000 l Sauerstoff und Druckluft. Sieben Einspeisungspunkte wurden mit je zwei 50-l-Flaschen Sauerstoff und Druckluft bestückt, mit einer Gesamtverfügbarkeit von je 175.000 l der beiden Gase. Je 8 weitere Flaschen waren zusätzlich in Reserve. Die Maßnahme wurde an einem Samstag ohne Elektivoperationsprogramm durchgeführt, sodass die betroffenen OP gesperrt werden konnten. Der Zeitpunkt wurde so gewählt, dass während des Nachmittagsschichtwechsels die doppelte Besetzung des Intensivpflegepersonals verfügbar war. Im Vorfeld wurden möglichst viele der Beatmungspatienten klinikintern verlegt. Neun Beatmungspatienten mussten allerdings verbleiben. Der technische Eingriff in die Gasversorgung dauerte lediglich 2 h, ohne Beeinflussung des Patientenzustands. Während der 2‑stündigen Interimsversorgung wurden auf den High-Care-Stationen 16.500 l Druckluft und 8000 l Sauerstoff verbraucht. Pro Beatmungspatient ergab sich rechnerisch ein Stundenverbrauch von 917 l Luft (15 l/min) und von 444 l Sauerstoff (7 l/min). Die vorausberechnete Menge auf Basis intensivmedizinischer Erfahrungswerte war deutlich geringer. Die 10fache Vorhaltung der Gasmenge und die vorhersehbar geringere Anzahl von Beatmungspatienten als die zugrunde gelegte Maximalbelegung haben dies mehr als kompensiert.

**Schlussfolgerung:**

Für technische Eingriffe in Hochrisikobereichen ist eine sorgfältige Planung und Durchführung in einem effektiven Team erforderlich. Etablierte Verfahren des Projektmanagements und der Risikobewertung helfen in der Fehlervermeidung.

## Abstract (en)

**Background:**

In the course of building extension works at Dresden University Hospital, it was necessary to shut down the central medical gas supply in a building with 3 intensive care wards with 22 beds, an operating theater tract with 5 operating rooms and 6 normal wards each with 28 beds during ongoing services. Thus, for the construction phase there was a need to establish an interim decentralized gas supply with zero failure tolerance for the affected functional units .

**Methods:**

Following established procedures for possible risk and failure analysis, a project group was set up by the hospital’s emergency and disaster management officer to develop a project plan, a needs assessment and a communication plan.

**Results:**

A variety of risk factors were systematically identified for which appropriate countermeasures needed to be designed. The needs assessment over 4 h based on physiological parameters for the maximum available 22 ventilator beds resulted in 26,000 l of oxygen and 26,000 l of compressed air. A total of 7 supply points were each equipped with two 50l cylinders for both oxygen and compressed air, with a total availability of 175,000 l of each of the 2 gases. Another eight cylinders each were held in reserve. The project was carried out on a Saturday without an elective surgery program, so that the operating rooms concerned could be closed. The timing was chosen so that double staffing of intensive care personnel was available during the afternoon shift change. In advance, as many of the patients on mechanical ventilation as possible were transferred within the hospital; however, nine of the mechanically ventilated patients had to remain. The technical intervention in the gas supply lasted only 2 h without affecting the patient’s condition. During the 2‑h interim supply, 16,500 l of compressed air and 8000 l of oxygen were consumed on the high-care wards. The calculated hourly consumption per ventilated patient was 917 l of air (15 l/min) and 444 l of oxygen (7 l/min). The quantity framework based on empirical values from intensive care medicine was significantly lower. This was more than compensated for by the 10-fold stocking of gas and the predictably lower number of ventilated patients than the maximum occupancy used as a basis.

**Conclusion:**

For technical interventions in high-risk areas, careful planning and execution in an effective team is required. Established procedures of project management and risk assessment help to avoid errors.

## Einleitung

Baumaßnahmen im Betrieb eines Krankenhauses haben meist einen indirekten oder direkten Einfluss auf die Krankenhausfunktionalität, ggf. seine Kapazität und auch auf medizinische Behandlungsprozesse [1]. Nicht nur die Implementierung neuester Technik, sondern auch die Instandhaltung und Erweiterung der Betriebstechnik sind wichtige Aspekte in der Fortentwicklung eines Krankenhauses [2].

**Figure 1:**
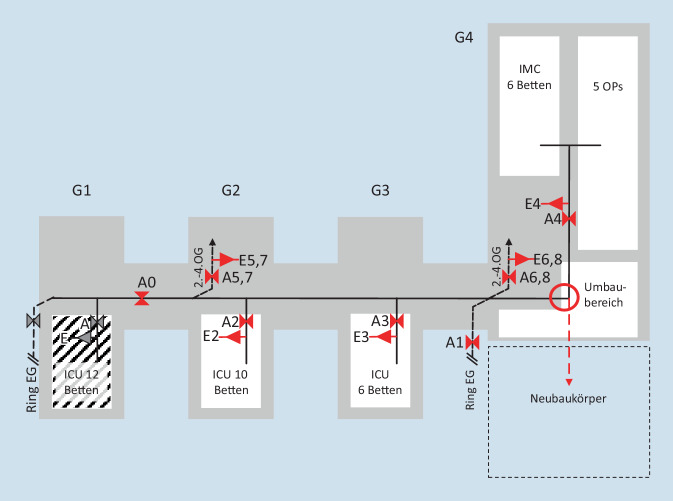

Im Rahmen von Erweiterungsbaumaßnahmen am Uniklinikum Dresden war eine vorübergehende Überbrückung der medizinischen Gasversorgung in Teilen des chirurgischen Zentrums notwendig. Daten zur Bemessung der Vorhaltungen für einen derartigen Eingriff sind bislang nicht publiziert. Die Umbaumaßnahme erfolgte im Gebäudeteil 4 (Abb. 1, *Kreis*).

Grundsätzlich wird das Gebäude an 2 Stellen über eine Ringleitung redundant eingespeist (Abb. 1, *Ring EG*). Die direkte Verbindung der Ringleitungseinspeisungen konnte nur zwischen den Gebäudeteilen 1 und 2 getrennt werden (Abb. 1, Markierung *A0*). Die Unterbrechung und Einspeisung ist aber auf der Stationsebene bzw. für den OP-Bereich selektiv möglich (Abb. 1, Markierungen *A1–A8*). Das abtrennbare Gebäudeteil 1 konnte während der Maßnahme weiter aus der Ringleitung gespeist werden. Für die Gebäudeteile 2–4, in denen 3 intensivmedizinische Teilstationen und 6 Normalstationen mit je 28 Betten untergebracht waren, bestand der Bedarf einer interimistischen dezentralen Gasversorgung. Aufgrund der im Wochenendbetrieb verfügbaren OP-Kapazitäten wurde der ebenfalls betroffene OP-Trakt für die Dauer der Umschlussmaßnahme außer Betrieb genommen. Selbst unter der Voraussetzung, dass die Patientensicherheit zu 100 % gewährlistet werden sein muss, ist eine mit 4 h veranschlagte Baumaßnahme, auch in einem medizinischen Hochrisikobereich, keine Rechtfertigung für eine komplette Unterbrechung des chirurgischen Krankenhausbetriebs; dies gilt insbesondere in der komplexen Versorgung an einem Universitätsklinikum. Ein komplettes Freiziehen aller betroffenen Bettplätze und die sukzessive Wiederbelegung nach der Maßnahme hätten eine überregional relevante Einbuße von spezialisierter chirurgischer Behandlungskapazität über mehrere Tage bedeutet [2]. Daneben müssen Nutzen und Risiken einer kompletten Patientenverlagerung, insbesondere von High-Care-Patienten in andere Klinikteile oder gar nach extern, genau erwogen werden [1, 3]. Im Sinne des konsequenzbasierten Modells der Krankenhausalarm- und Krankenhauseinsatzplanung nach Wurmb [1] handelte es sich im vorliegenden Szenario um einen Eingriff in die Krankenhausbetriebstechnik mit potenziellen Folgen für die Funktionalität der Klinik und in der Konsequenz ihrer Kapazität. Eine ausreichende Vorbereitungszeit ohne Bestehen einer Eigengefährdung war gegeben.

Kommunikationsmängel sind die Hauptursache für Fehler in medizinischen Hochrisikobereichen [4]. Entsprechend wurde zur Risikoabschätzung und Projektabstimmung der Beauftragte für das Notfallmanagement und den Katastrophenschutz des Uniklinikums involviert [5]; dieser stellte in seinen weiteren Rollen als Sprecher der OP-Steuergruppe und leitender Oberarzt in der Anästhesie eine interprofessionelle Projektgruppe aus Technikern, Klinikern, Pflegeleitungen sowie dem medizinischen Qualitäts- und Risikomanagement zusammen.

Ziel war, eine Umsetzungsmöglichkeit mit Nullfehlertoleranz zu schaffen [6, 7]. Dazu gehört sowohl eine Bedarfsabschätzung als auch eine Fehlermöglichkeitsanalyse [8, 9], um mögliche Gefahrenquellen im Vorfeld erkennen sowie den Prozess für alle Beteiligten verständlich und sicher planen zu können. Ein wichtiger Erfolgsfaktor ist die direkte Einbindung aller am Prozess beteiligten Fachdisziplinen und Berufsgruppen [7, 10, 11]. Höchste Priorität in der Vorbereitung der Maßnahme hatte deshalb die enge Abstimmung zwischen den medizinischen Abläufen sowie der technischen und personellen Absicherung in der Durchführung. Mithilfe der Error-Risk-Analyse sollten mögliche Fehlerquellen im Vorfeld aufgedeckt werden [8, 12].

## Error-Risk-Analyse

Die Error-Risk-Analyse nach dem London-Protokoll ist ein Instrument des klinischen Risikomanagements [8, 12]. Mithilfe eines strukturierten Verfahrens stellt es ein Modell dar, das sich auf die systematische und organisatorische Unfall‑/Fehlerentstehung konzentriert und in seiner retrospektiven Anwendung nicht auf Schuldzuweisungen basiert [12]. Aufgrund von äußeren Einflüssen und bewusst bzw. unbewusst durchgeführten Handlungen können Fehler entstehen und Patienten gefährdet werden [13]. Auch müssen Maßnahmen ergriffen werden, damit ggf. bestehende Konflikte der Akteure keine Auswirkung auf den Erfolg der Maßnahme und insbesondere die Patientensicherheit haben [9, 14].

Es lassen sich aktive und latente Fehler unterscheiden, die zudem von variablen Einflussfaktoren unterstützt werden [12, 15]. Aktive Fehler sind aktive Handlungen oder Unterlassungen, die routinemäßig, situations- und einzelfallbedingt auftreten. Hierzu zählen fertigkeits-, entscheidungs- oder wahrnehmungsbasierte Fehler. Latente Fehler hingegen sind aggravierende Begleitumstände, die erst in der Koinzidenz mit aktiven Fehlern ihre Wirkung entfalten [12, 16]. Sie werden üblicherweise erst erkannt, wenn das unerwünschte Ereignis bereits eingetreten ist [17], sind aber folglich hierfür nicht allein verantwortlich [9]. Dennoch kann nach jenen Faktoren gezielt gefahndet werden, bevor eine besondere Maßnahme, wie der im vorliegenden Beitrag betrachtete Gasumschluss im laufenden Betrieb, durchgeführt wird.

## Projektplanung

In einem Gebäudeteil des Universitätsklinikum Dresden (UKD; Abb. 1, Kreis) sollte die medizinische Gasversorgung im zentralen Leitungsnetz unterbrochen werden, um den Anschluss eines Erweiterungsbaus zu ermöglichen. Zur unterbrechungsfreien medizinischen Gasversorgung wurde eine Interimsversorgung mit Gasflaschen geplant. Im Vorfeld des Projekts wurde vorgesehen, in den vom flaschengestützten Interimsbetrieb betroffenen Intensivstationsabschnitten der Gebäudeteile 2–4 (Abb. 1) möglichst wenige Beatmungspatienten zu belassen. Dadurch wurden der Gasverbrauch möglichst gering gehalten sowie die Fehlermöglichkeiten in der Logistikkette der Gasflaschen und bei ihrem Wechsel im laufenden Interimsbetrieb reduziert [1]. Ebenso wurde entschieden, dass an allen 7 Einspeisungspunkten 50 l Gasflaschen zum Einsatz kommen, um Flaschenwechsel im Interimsbetrieb auf ein Minimalmaß zu begrenzen. Identisch aufgebaute Einspeisungspunkte erleichtern zudem deren Interoperabilität mit Material und Personal und reduzieren Fehlermöglichkeiten [7, 9].

Beatmungspatienten wurden am Vortag, soweit möglich, in das Gebäudeteil 1 mit separatem Gasnetzanschluss bzw. in andere Intensivstationen des UKD aufgenommen bzw. verlegt. Um einen zusätzlichen Gasbedarf aus dem OP-Bereich zu vermeiden, erfolgte die Maßnahme an einem Samstag, an dem kein Elektivprogramm in den betroffenen 5 OP geplant worden war. Das Notfallprogramm konnte in anderen nichtbetroffenen Gebäudeteilen des Zentral-OP abgewickelt werden. Eine Bedarfsplanung wurde gemeinsam mit der Medizintechnik und dem Notfallmanagement der Klinik als Worst-Case-Szenario mit Beatmungsvollauslastung durchgeführt. Aus der Maximalbelegung der Stationsteile mit Beatmungspatienten und der Dauer der Maßnahme von maximal 4 h wurde der maximal zu erwartende Gasbedarf unter Zugrundelegung üblicher Beatmungsparameter ermittelt (Tab. 1).

**Table 1:** 

	Sauerstoff (l)	Druckluft (l)
**Bedarfsschätzung**		
Pro Beatmungspatient und min	5	5
Pro Beatmungspatient und 4 h	1.200	1.200
*Pro Station über 4* *h*		
ICU, G2, 10 Plätze	12.000	12.000
ICU, G3, 6 Plätze	7.200	7.200
IMC, G4, 6 Plätze	7.200	7.200
*Medizinische Gesamtschätzung*	*26.400*	*26.400*
**Bereitstellung**		
Technische Bereitstellung, High Care	75.000	75.000
Technische Bereitstellung, Low Care	100.000	100.000
Reserve vor Ort	100.000	100.000
*Verfügbarkeit, gesamt*	*275.000*	*275.000*
**Tatsächlicher Verbrauch über 2** **h Interimsbetrieb**		
Verbrauch, High Care	8000	16.500
Verbrauch, Low Care	2.000	1.000
*Verbrauch, gesamt*	*10.000*	*17.500*

*AMV* Atemminutenvolumen, *F*_*I*_*O*_*2*_ inspiratorische Sauerstofffraktion, *ICU* Intensive Care Unit

Es wurde ein Szenario mit invasiver Beatmung mithilfe der gasdruckbetriebenen Beatmungsgeräte EVITA 4 (Fa. Dräger, Lübeck [18]) kalkuliert. Entsprechend den bestehenden Erfahrungswerten bei Beatmungen ohne nichtinvasive Form („noninvasive ventilation“, NIV) oder High-Flow-Nasenkanülen („nasal high-flow cannula“, NHFC) wurden ein Atemminutenvolumen (AMV) von 6,5 l, eine durchschnittliche inspiratorische Sauerstofffraktion (F_I_O_2_) von 0,5 und ein Gasverbrauch für den Gerätebetrieb von 3,5 l/min zugrunde gelegt [18]. Der Sauerstoffbedarf für Insufflationen in den restlichen Gebäudeteilen wurde durch den im Wesentlich technisch bedingten 10fachen Sicherheitspuffer mitberücksichtigt; dieser sollte die Möglichkeit der zeitlichen Ablaufverzögerung ebenso abbilden wie Abweichungen des tatsächlichen vom geschätzten Verbrauch. Da die Vollauslastung aller Beatmungskapazitäten im betroffenen Bereich nach Erfahrungswerten nicht zu erwarten war und auch im Bedarfsfall Verschiebungen in das Gebäudeteil 1 sowie auf andere Stationen mit Beatmungskapazität möglich waren, erschien die Vorhaltung entsprechend Tab. 1 den realistisch erwartbaren Bedarf um mehr als das 10Fache zu übersteigen.

Die Umbaumaßnahme wurde mit einem Vorlauf von 4 Wochen geplant, und alle betroffenen Mitarbeitenden wurden informiert [19]. Um als Rückfallebene für ein nichtvorhersehbares Ausfallszenario ausreichend Kräfte zur Verfügung zu haben, wurde als Zeitpunkt der überlappende Nachmittagsschichtwechsel der Intensivpflegekräfte gewählt, und Transportbeatmungsgeräte wurden vorgehalten. Im Rahmen eines Ausfallszenarios hätten Beatmungspatienten in näher gelegene, am Wochenende ungenutzte Aufwachräume oder OP, die von der Gasabschaltung nicht betroffen waren, verbracht werden können.

Für jeden der Einspeisungspunkte wurde ein verantwortlicher Techniker eingesetzt, ebenso war ein redundanter Flaschentransportdienst verfügbar. Eine Kommunikationsliste (Tab. 2) mit allen Beteiligten ermöglichte die schnelle Erreichbarkeit über Digital Enhanced Cordless Telecommunications (DECT) zwischen allen internen und externen Kräften sowie den Verantwortungsträgern in der Klinik [19]. Dadurch ist eine klare Rollenverteilung definiert. Die zentrale Koordination wurde gemeinsam von einem Ingenieur und dem medizinischen Katastrophenschutzbeauftragten der Klinik wahrgenommen [19]. Eine Bettenabmeldung bei der Rettungsleitstelle erfolgte für die Maßnahme nicht, da entsprechender Kompensationsspielraum in anderen Klinikbereichen existierte.

**Table 2:** 

Etagenverteiler	Station	Ansprechpartner med. Versorgung (Tel.-Nr.)	Techniker (Tel.-Nr.)	O_2_ (50 l)	AIR (50 l)
–	–	Leitender Ingenieur	*[DECT]*	–	–
–	–	KatS Beauftragter (S2/S3)	*[DECT]*	–	–
–	–	Pflegedienstleitung (S1/S4)	*[DECT]*	–	–
E1	Ambulanz	Am Wochenende geschlossen	Sperrung	–	–
E2	ITS G2	1 Oberarzt *[DECT]* 1 Facharzt *[DECT]*	A *[DECT]*	2	2
E3	ITS G3		B *[DECT]*	2	2
E4	IMC G4	Stationsleitung *[DECT]*	C *[DECT]*	2	2
E5	Wachstation	Stationsleitung *[DECT]*	D *[DECT]*	2	2
E6	Normalstation	Stationsleitung *[DECT]*	E *[DECT]*	2	2
E7/7′	2 Normalstationen	Pflegekraft *[DECT]*	F *[DECT]*	2	2
E8/8′	2 Normalstationen	Pflegekraft *[DECT]*	G *[DECT]*	2	2
OP	OP-Teilbereich	Anästhesie *[DECT]*/OP *[DECT]*	Sperrung	–	–
Reserve	Keller	Transportdienst *[DECT]*	–	8	8

*AIR* Druckluft, *DECT* Digital Enhanced Cordless Telecommunications, *KatS* Katastrophenschutz

## Projektdurchführung

**Figure 2:**
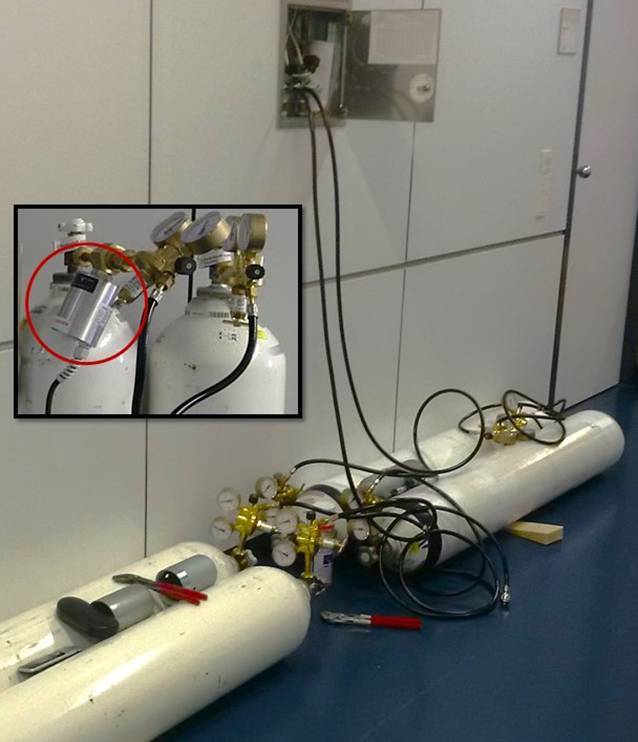

Zwei Stunden vor der geplanten Einspeisung wurden die Gasflaschen und das zugehörige Equipment an die Einspeisungspunkte transportiert. Daraufhin erfolgten die Montage der Interimsversorgung an den Etagenverteilern (Abb. 2) sowie die Funktionsprüfung der Anschlussgeweihe. Die Versorgungsbatterien wurden mit 50 l Gasflaschen in Zwillingsmontage realisiert, um einen Austausch der Flaschen im Betrieb zu ermöglichen. Eine standardmäßig nicht vorhandene Aufrüstung mit Sauerstoff- und Druckluftflaschen an allen Bettplätzen wurde nicht für notwendig erachtet, da von der Versorgungsstabilität über die interimistische dezentrale Gasversorgung ausgegangen wurde.

In Erwartung hoher Durchflussraten an den Flaschenventilen wurden entsprechende Wärmeelemente (Bildausschnitt) eingebaut, die die Vereisung der Ventile bzw. Anschlussgewinde zuverlässig verhinderten. Vereiste Ventile bzw. Gewinde erschweren einen Flaschenwechsel erheblich oder verhindern ihn gar. Neben den jeweils vorgehaltenen 275.000 l medizinischen Sauerstoffs und der Druckluft wurde der externe Lieferant für medizinische Gase für den Tag der Maßnahme vertraglich gebunden, falls notwendig, weiteren Bedarf durch sofortige Zulieferung zu decken.

Die technischen und organisatorischen Vorbereitungen waren planmäßig vor dem Schichtwechsel der Intensivstationen abgeschlossen. Nach Eintreffen der Spätschicht der Intensivpflege auf den Teilstationen und letztem Briefing um 14 Uhr erfolgten die abschnittsweise Absperrung der zentralen Gasversorgung und die Prüfung der Versorgungsstabilität durch die dezentralen Flaschenbatterien in einer vorbestimmten Reihenfolge. Bei stabilen 5 bar kam es in keinem Fall zu Druckschwankungen. Die Versorgungsumstellung an den Bettplätzen vollzog sich entsprechend unmerklich stabil. Die Abschaltung der Steigleitungen erfolgte im Fünfminutentakt, wobei ein Absperrventil nur mithilfe einer Zange geöffnet werden konnte. Der Zugang zu einem anderen Absperrventil wurde verzögert, weil sich Elemente der Hängedecke nicht entfernen ließen. Zwanzig Minuten später wurde im Gesamtsystem als „point of no return“ [7, 17] der Druck abgelassen, sodass die Umbauarbeiten planmäßig durchgeführt werden konnten.

Blutgasanalysen der beatmeten Patienten vor und während des Umschlusses zeigten keine Auswirkungen durch den temporären dezentralen Flaschenbetrieb.

Bereits um 16 Uhr waren die Arbeiten mit der Rückschaltung zur zentralen Gasversorgung abgeschlossen. Danach wurden die medizinischen Verantwortungsbereiche informiert, und die Fertigstellung wurde übermittelt. Die Interimsversorgung konnte dementsprechend demontiert und der Baubereich gereinigt werden.

Der Intensivbereich im Gebäudeteil 1 blieb unbeeinflusst in Betrieb. Die betroffenen OP blieben für den Zeitraum der Umbauarbeiten gesperrt, bis die Freigabe durch die Techniker erfolgt war. Notwendige operative Eingriffe im Umschlusszeitraum wurden in OP-Bereichen anderer Gebäudeteile durchgeführt.

## Diskussion

Krankenhäuser jeglicher Größe sind hochkomplexe Einrichtungen der Daseinsvorsorge [11, 20]. Eine Beeinträchtigung der Betriebssicherheit stellt grundsätzlich eine Gefährdung der anvertrauten Patienten dar, weshalb Systeme mehrfach abgesichert sein müssen. Gleichzeitig entwickelt sich die Medizin ständig weiter, sodass auch medizinische Einrichtungen mit dem Fortschritt u. a. baulich Schritt halten müssen. Ein „Abmelden“ von der medizinischen Versorgung für geplante Umbaumaßnahmen ist ab einer bestimmten Versorgungsstufe nicht möglich, ohne die überregionale Versorgung mit spezialisierten Leistungen zu beeinträchtigen [2, 21]. Dies bedingt u. a., dass Baumaßnahmen im laufenden Betrieb durchgeführt werden müssen. Die Bedeutung dieses Zusammenspiels im Patientenversorgungsbetrieb ist vielen Mitarbeitenden im Gesundheitswesen nicht bewusst. Ein entsprechendes Bewusstsein fördern Maßnahmen des Qualitäts- und Risikomanagements, z. B. in Form der Durchführung von internen bzw. Risiko-Audits [8].

Am UKD wurde im Rahmen von Erweiterungsbaumaßnahmen die Abschaltung der zentralen Gasversorgung in einem Gebäude mit 34 High-Care- bzw. Intermediate-Care-Betten, 5 OP und 6 Normalstationen mit insgesamt 168 Betten während des laufenden Krankenhausbetriebes erforderlich. Daten zum Gasverbrauch eines derartigen Eingriffs in den Krankenhausbetrieb, insbesondere für eine dezentrale Flaschengasversorgung, sind bislang nicht publiziert.

Die Umbaumaßnahme wurde durch eine interprofessionelle Projektgruppe geplant und begleitet; diese sollte sicherstellen, dass eine Patientengefährdung ausgeschlossen wird [9, 22, 23]. Insgesamt wurde von den vorgehaltenen 550.000 l der medizinischen Gase in 50-l-Flaschen nur rund ein Zwanzigstel, genau 27.500 l verbraucht. Für die Intensivstationen mit insgesamt noch 9 Beatmungspatienten während der 2‑stündigen Interimsversorgung zeigte sich ein Verbrauch der Druckluft von 16.500 l und des Sauerstoffs von 8.000 l. Die sich hieraus ergebende technische F_I_O_2_ von 33 % ist unter Berücksichtigung der Ablesetoleranz der analogen Flaschenmanometer plausibel nahe bei den mittleren automatisiert im Patientendatenmanagementsystem (PDMS) dokumentierten Einstellungen der Beatmungsgeräte von 37 %. Damit kann für die betreute Patientenklientel pro Beatmungspatient ein Stundenverbrauch von 917 l Luft (15 l/min) und von 444 l Sauerstoff (7 l/min) errechnet werden. Die in der Planung verwendeten Schätzwerte, die sich an physiologischen Größen orientiert haben (Tab. 1), lagen deutlich darunter. Nicht berücksichtigt, da nicht sicher bestimmbar, war das Gasvolumen der Gebäudegasversorgungsanlage. Der 10fache Sicherheitsaufschlag in der vorgehaltenen Gasmenge gegenüber der Bedarfsschätzung und die nach Erfahrungswerten vorhersehbar deutlich geringere Anzahl von Beatmungspatienten (*n* = 9) haben dies mehr als kompensiert. Der Bedarfsberechnung wurde absichtlich eine praktisch auszuschließende Maximalbelegung mit 22 Beatmungspatienten zugrunde gelegt.

Ein Umstand muss im Zusammenhang mit der Versorgung aller Abschnitte mit den festen Mengengebinden der 50-l-Flaschen (Tab. 2) berücksichtigt werden: Die Low-Care-Abschnitte E5–E8 haben mit ihrem geringem Verbrauch von 3.000 l in 2 h eine anderweitig nicht nutzbare Gaskapazität von 200.000 l gebunden. Damit ist bei der Festlegung der vorzuhaltenden Gasmenge die Kapazitätsbindung bei Kleinverbrauchern zu ergänzen. Im Worst Case könnte ein Ringtausch dieser Flaschen unter Einbindung der Flaschenreserve erwogen werden, um Bereiche mit hohem Verbrauch nachzubestücken.

Durch die systematische technische und organisatorische Vorbereitung [12], die Verfügbarkeit von Führungskräften vor Ort [19] sowie die Gemeinschaftsleistung und das Engagement aller Mitarbeitenden sind keine unerwarteten Ereignisse eingetreten. Das Aufrufen des Krankenhausalarm- und Krankenhauseinsatzplans mit vollständiger Vorhaltung der Krankenhauseinsatzleitung [11, 24, 25] für eine solche Maßnahme muss dem Nutzen gegenübergestellt werden. Im Sinne der Stabsfunktionen nach Feuerwehrdienstvorschrift (DV 100 [26]) war lageangepasst ein verkleinerter Stab mit den zusammengelegten Funktionen Lage (S2) und Einsatz (S3) durch den Katastrophenschutzbeauftragten vor Ort vertreten. Die Stabsfunktionen Personal (S1) und Logistik (S4) wurden durch die diensthabende Pflegedienstleitung abgebildet und hätten lageelastisch weiter entfaltet werden können [5, 27]. Unabhängig davon kann ein solches Szenario je nach Erfahrungsgrad des jeweiligen Krankenhauses mit stabsmäßiger Führung genutzt werden, um die Einsatzerfahrung der Klinikeinsatzleitung zu fördern [5, 24, 25].

Entscheidend für die technische Planung ist das Wissen um die kompletten Gaslaufpläne. Bereits in der Planungsphase von Krankenhausgebäuden ist dafür zu sorgen, dass genügend Absperrmöglichkeiten in alle Infrastrukturnetze (Gase; Wasser, Elektrizität usw.) integriert werden. Unbekannte, nicht in Bauplänen verzeichnete Ventile oder Einspeisungen können dazu führen, dass ein redundanter Zufluss verhindert, dass Drucklosigkeit eines Systems für Umbaumaßnahmen hergestellt werden kann. Andererseits hätten bauseits verfügbare Gasabsperrventile analog dem Ventil *A0* (Abb. 1) zwischen den Gebäudeteilen 2 und 3 bzw. zwischen 3 und 4 eine Abwicklung der Umschlussmaßnahme mit nur einem Drittel der betroffenen Patientenbereiche ermöglicht. In diesem Sinne kann die fehlende Einplanung dieser beiden Absperrventile als latente Fehlermöglichkeit, die sich erst Jahrzehnte später auswirkt, noch aus der Bauplanung herrührend interpretiert werden [9, 13].

## Vorbeugendes Risikomanagement

Fehlerbegünstigte Faktoren, die vor der Durchführung der Gasumstellung im Rahmen des Risiko- und Patientensicherheitsmanagements betrachtet werden mussten, sind in Tab. 3 aufgeführt. Zentraler Punkt ist die 100 %ige Gewährleistung der Patientensicherheit, trotz fehlender vergleichbarer Erfahrungen oder publizierter Berichte. Der planbare Charakter der Maßnahme mit potenzieller Auswirkung auf Funktionalität und nachfolgend die Kapazität des Krankenhauses [1] erhöht den Druck auf eine fehlerfreie Durchführung. Allgemeine Erfahrungswerte aus Anästhesie und Intensivmedizin können auf mögliche Gefahrenquellen hinweisen.

**Table 3:** 

Faktorart	Einflussnehmende Faktoren
Patientenfaktoren	Zustand (Komplexität und Schweregrad) Sprache und Kommunikation Persönlichkeit und soziale Faktoren
Aufgaben- und Verfahrensfaktoren	Aufgaben- und Prozessgestaltung sowie strukturelle Klarheit Verfügbarkeit und Verwendung von Richtlinien und Verfahrensanweisungen Verfügbarkeit und Genauigkeit von Testergebnissen Entscheidungshilfen
Individuelle Faktoren (Personal)	Kenntnisse und Fähigkeiten, Kompetenz Körperliche und psychische Gesundheit
Teamfaktoren	Mündliche Kommunikation Schriftliche Kommunikation Supervision und Hilfesuche Teamstruktur (Passung/Übereinstimmung, Beständigkeit, Führung usw.)
Faktoren des Arbeitsumfeldes	Personalbestand und Qualifikationsmix Arbeitsbelastung und Schichtpläne Beschaffenheit, Verfügbarkeit und Instandhaltung der technischen Ausstattung Unterstützung durch Verwaltung und Geschäftsleitung Physische Umgebung
Organisation und Managementfaktoren	Finanzielle Ressourcen und Einschränkungen Organisationsstruktur Grundsätze, Standards und Ziele Sicherheitskultur und Prioritäten
Faktoren des institutionellen Rahmens	Wirtschaftlicher und regulatorischer Kontext Gesundheitspolitik Verbindungen mit externen Organisationen

Finanzielle Grenzen dürfen die Patientensicherheit nicht einschränken, werden aber durch die Organisation selbst gesetzt. Im vorliegenden Fall waren Kostenaspekte in der personellen und materiellen Ausstattung des Projekts nicht relevant. Um allerdings die Risiken zu minimieren, ist die Maßnahme in kürzest möglicher Zeit zu realisieren. Aufgrund paralleler Anwesenheit steigen zwar die Personalkosten, gleichzeitig reduzieren sich aber der Bedarf an Flaschengas mit entsprechender Logistik und die Planungsunsicherheit in der Bemessung des Bedarfs. Diese Intervallverkürzung zwingt zur technisch bestens vorbereiteten Durchführung des Umbauprozesses.

Unvollständige oder nichtverständliche Protokolle können ebenso Fehler begünstigen. Die unzureichende Kommunikation zwischen den verschiedenen Berufsgruppen (medizinisches Personal, interne und externe Techniker) kann zu Informationsverlusten führen; gelingende Kommunikation ist ein Schlüsselfaktor zur Fehlervermeidung in hochkomplexen Systemen [7]. Effektives Nachfragen und Wiederholen des Gesagten sind für ein gleiches Verständnis der Prozessschritte besonders hilfreich [28]. Besonders medizinische Mitarbeitende ohne Leitungsfunktion müssen in die bevorstehenden Maßnahmen eingewiesen und bei deren Umsetzung unterstützt werden [4, 9]. Vor allem Beobachtungen an medizintechnischen Geräten oder gar von Zustandsveränderungen der Patienten während der Umbaumaßnahme müssen schnellstens an das ärztliche Personal kommuniziert werden. Wenn der Verdacht des Zusammenhangs mit der Umbaumaßnahme besteht, muss die Projektleitung umgehend informiert werden, um Abhilfe zu schaffen [19].

**Figure 3:**
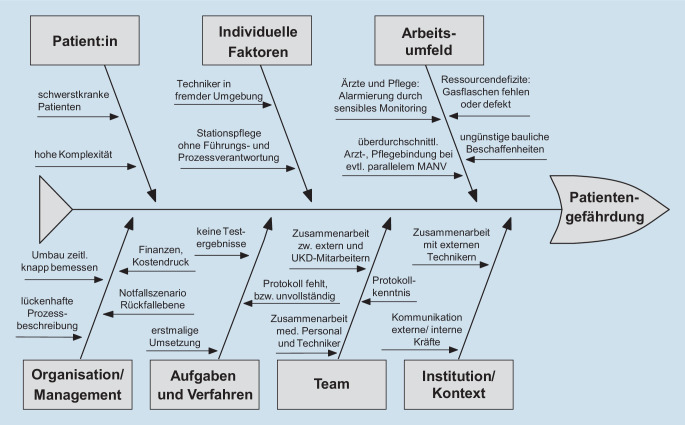

Ebenso sollten jene Mitarbeitenden Unsicherheiten ohne Ängste der Zurückweisung mitteilen können [9, 16]. Eine weitere Herausforderung ist die Zusammenarbeit mit dem Personal eines externen Dienstleisters, das die Umbaumaßnahmen vornimmt. Dieses kennt die baulichen und klinikorganisatorischen Strukturen nicht in dem Maße wie das interne Personal, weshalb eine sehr enge Absprache notwendig ist. Eine Zusammenfassung der Risikofaktoren zeigt das Ishikawa-Diagramm (Abb. 3).

**Figure 4:**
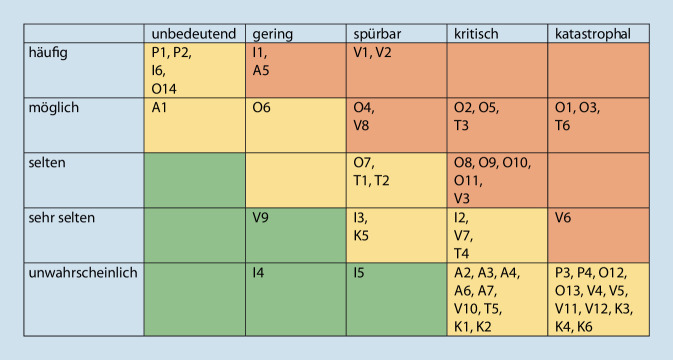

**Figure 5:**
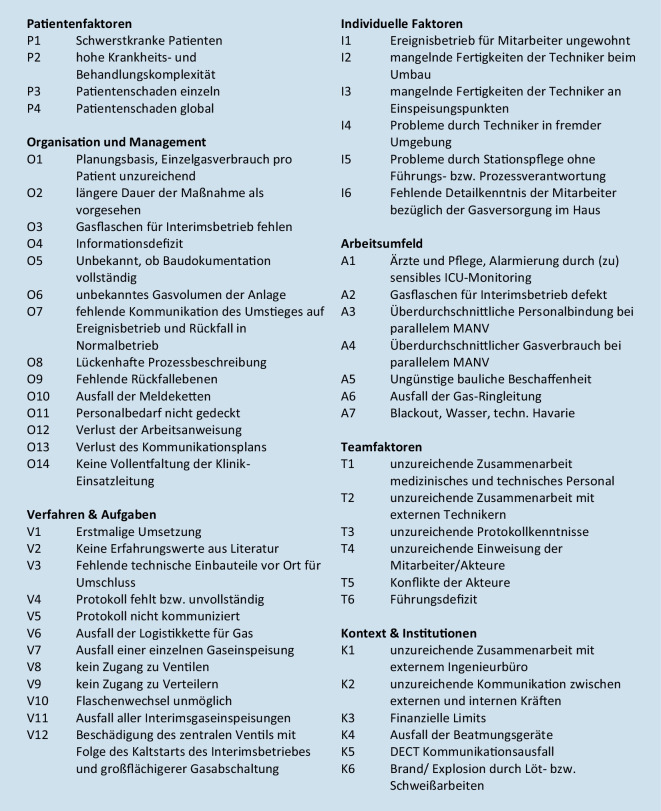

Ein Ishikawa-Diagramm ist zwar gut geeignet, um Risikofaktoren darzustellen, nicht aber, um diese zu quantifizieren. Quantitative Aspekte kann eine Risikolandschaft (Abb. 4) gut visualisieren [8]. Die zugrunde liegende Bewertung ist zwar im Organisationskontext immer auch in gewisser Weise variabel und subjektiv, erlaubt aber dennoch eine Gewichtung, welchen Risiken in besonderer Weise begegnet werden muss. Auf die Berechnung der hieraus abgeleiteten Risikoprioritätenzahl (RPZ) wurde im vorliegenden Kontext bewusst verzichtet, da der dritte Faktor, die Detektionswahrscheinlichkeit (D) neben der beschriebenen Eintrittswahrscheinlichkeit (W) und dem Schadenausmaß (A) für alle in der Projektgruppe bereits detektierten Risiken (Abb. 5) als konstant anzunehmen ist. Nichtdetektierte Risiken dieser Maßnahme konnten entsprechend auch nicht bewertet werden.

$$\mathrm{RPZ}=\mathrm{W\ }\cdot \mathrm{A\ }\cdot \mathrm{D}$$

(Maximal 10 • 10 • 10 = 1000, mindestens 1 • 1 • 1 = 1, [8]).

**Figure 6:**
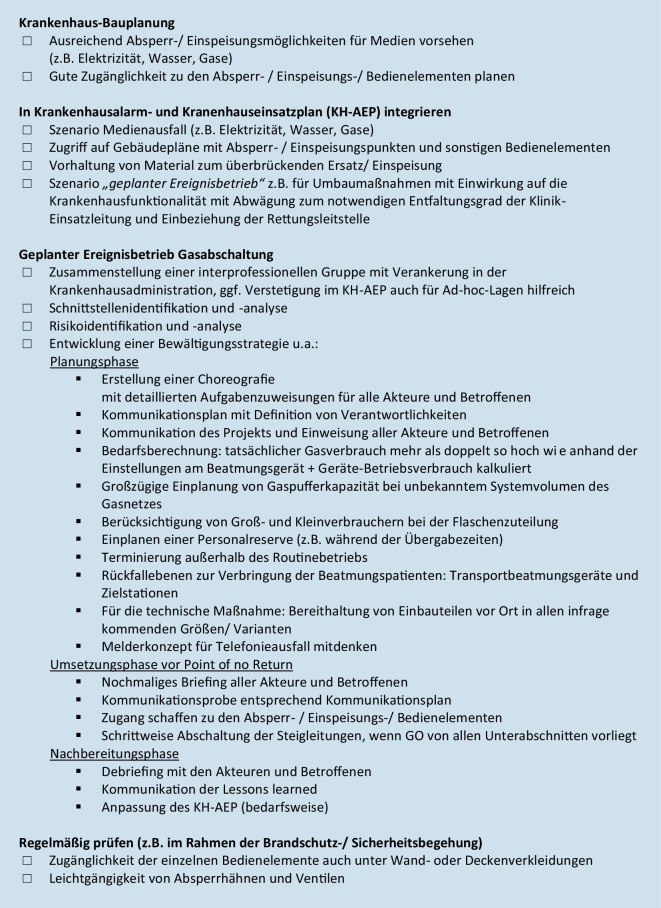

Entsprechend der Bewertung der erkannten Risiken ist insbesondere denjenigen in der rechten oberen Ecke von Abb. 4 (*rot*) entgegenzuwirken. Hier mussten zuvorderst die fehlerhafte Gasmengenplanung (*O1*), fehlende Gasvorhaltung (*O3*) und eine unzureichende Führung (*T6*) bei der Bewältigungsplanung in den Fokus genommen werden.

## Risikomanagement am Beispiel

Das Beispiel der Umbaumaßnahmen des Gasversorgungssystems von Intensivstationen konnte deutliche Fehlerquellen aufzeigen. Die Ergebnisse des Gasverbrauches haben gezeigt, dass vorab anhand physiologischer Größen mit 10 l/min und Bettplatz zu wenig Gas bemessen wurde. Wären anstatt der 9 – wenngleich ausgesprochen unwahrscheinlich – tatsächlich 22 Patienten beatmet worden, hätte auf die Reserve zurückgegriffen werden müssen (Tab. 1 und 2), um in den Abschnitten E2 und E3 je eine Druckluftflasche zu wechseln. Die Dauer der Umbauarbeiten wurde auf 3 bis 4 h geschätzt. Bei einem mittleren Verhältnis von Druckluft zu Sauerstoff von 1 zu 1 wurde ein Gasbedarf von 52.800 l geschätzt. Mit einem 10fachen Sicherheitspuffer wurden insgesamt 550.000 l Gas in Flaschen vor Ort zur Verfügung gestellt, einschließlich der Möglichkeit der zeitgerechten Nachlieferung bei absehbarer Verknappung. Insgesamt wurde an 9 Beatmungsplätzen 27.500 l Gas in 2 h Umbauphase verbraucht. Bei der gegebenen Belegung wäre eine Durchhaltefähigkeit von 12 h gewährleistet gewesen, mit Ringtausch der Flaschen der Kleinverbraucher von 33 h.

Weiterhin hätten fertigkeitsbasierte Fehler auftreten können, wenn eine hohe Arbeitsbelastung bzw. Überforderung bei Ausführen der Aufgabe entstanden wäre. Dies passiert u. a., wenn Mitarbeitende unter zeitlichem Druck stehen. Treten unerwartete Probleme in Form von technischen Hindernissen auf, kann Hektik bei der Arbeit ausbrechen. Im Fall der Deckenöffnung zu einer Gasleitung kam es zeitlich vor dem „Point of no return“ tatsächlich zur Umbauverzögerung, die sich lange hingezogen haben könnte. Der Fehler konnte schnell behoben werden, ist jedoch im Vorfeld der Planung einzubeziehen. Auch ärztliche und pflegerische Mitarbeitende können aufgrund verschiedener Einflussfaktoren den Überblick bei der Kontrolle der Patienten mit Umstellung der Gasversorgung verlieren. Ein unerwartet hoher Zustrom neuer Patienten, viele Zustandsverschlechterungen zu gleicher Zeit und das Auftreten eines unerwarteten Massenanfalls von Verletzten, der eine hohe Zahl der Ärzte und Pflegekräfte, aber auch der Führungspersonen bindet und Intensivbetten einfordert, sind einige Beispiele hierfür [1, 5]. Vorab wurde mit anderen Intensivbereichen abgestimmt, dass im Notfall eine Verlegung auf diese Stationen möglich ist. Ebenso hätten sog. Aussetzer entstehen können, wenn den Akteuren wichtige Informationen zum Ablauf oder zum Bauplan verloren gegangen wären. Hierzu gab es aber ein klares, allen ausgehändigtes Protokoll, einen Kommunikationsplan (Tab. 2) und jederzeit Ansprechpartner vor Ort.

Unabhängig von einer technischen Maßnahme ist es im Rahmen der Resilienzbildung für Systemausfälle in der kritischen Infrastruktur Krankenhaus [3, 11, 20] letztlich auch wichtig, dem Personal solche Erkenntnisse zu kommunizieren. Vielfach fehlt das Wissen darüber, wie die Sauerstoffversorgung eines Krankenhauses erfolgt, und wo technische Grenzen liegen. Abb. 6 fasst die gewonnenen Erfahrungen in einer Checkliste zusammen.

## Fazit für die Praxis

- Umbaumaßnahmen an der Gasversorgung einer Intensivstation erzeugen einen hohen organisatorischen Aufwand und bedürfen der detaillierten Vorausplanung unter Einbeziehung verschiedenster Expertisen.
- Oberstes Ziel ist, eine Patientengefährdung auszuschließen. Dies gelingt durch effektive Kommunikation aller beteiligten Personen und Berufsgruppen sowie durch Nutzung von Methoden des Projekt- und Risikomanagements.
- Vor der Maßnahme muss analysiert werden, welche Zwischenfälle bis hin zum Worst-Case-Szenario eintreten können und durch welche Rückfallebenen ihnen begegnet werden kann.
- Beispielsweise ist für die Vorhersage der benötigten Gasmengen eine alleinige Berechnung des Verbrauchs durch die Beatmung der Patienten nicht ausreichend. Vielmehr müssen u. a. das Füllungsvolumen von Gasleitungen in wenig genutzten Gebäudeteilen und die Kapazitätsbindung von Gasvolumina in den großen Flaschen der Kleinverbraucher mitberücksichtigt werden, da diese zur Versorgung der Beatmungspatienten primär nicht zur Verfügung stehen.
